# GenoITS: Implementation of an Integrated Testing Strategy workflow for genotoxicity using QSAR-based tools

**DOI:** 10.1016/j.namjnl.2024.100005

**Published:** 2024-12-28

**Authors:** José Luis Vallés-Pardo, Eva Serrano-Candelas, Addel Goya-Jorge, Salvador Moncho, Mar Crespo, Donna S Macmillan, Rafael Gozalbes

**Affiliations:** a ProtoQSAR S.L., Paterna, (València), Spain; b Toxiconsultant S.L., Nava, Spain; c Humane Society International, Washington D.C., WA, USA; d MolDrug AI Systems S.L., València, Spain

**Keywords:** QSAR, GENOTOXICITY, NAMs, ITS

## Abstract

To assess the potential impact of chemical substances on human health and the environment, and to ensure the safety of chemicals, the EU's REACH regulation requires registrants of chemicals to undertake a battery of tests to fulfil Standard Information Requirements. These tests raise two main concerns, (1) the economic burden due to the resources needed to perform the tests, both in materials and time invested, and (2) the ethical considerations of using animal testing. For this reason, nowadays the regulatory agencies encourage registrants to minimize the number of experiments performed on animals (3Rs). In this work we have developed an exclusively computational Integrated Testing Strategy to assess the genotoxicity of chemicals. A complete set of data of assays for gene mutation and cytogenotoxicity (in bacteria, *in vitro* and *in vivo*) are used. These assays are integrated into an automated workflow called GenoITS, which is part of the ProtoPRED^Ⓡ^ prediction suite (https://protopred.protoqsar.com/). This automated workflow is based on the schema proposed by REACH to assess the genotoxicity. The GenoITS workflow leads the user through this testing strategy, allowing to work with their own data and filling the gaps with our in-house QSAR (Quantitative Structure-Activity Relationship) models, and a binary classification result (genotoxic/not genotoxic) is delivered as a final output, by combining the results of the different tests required by different regulations to evaluate the genotoxicity. This workflow is presented as a commercial web-based technological platform that will provide significant benefit to the community using REACH and other regulations.

## Highlights

• Decisional workflow to assess the genotoxicity following REACH regulations.
• Assessment performed using experimental data and QSAR predictions.
• Output as a binary classification (genotoxic/non-genotoxic).
• Assessment report and QPRFs for each prediction automatically delivered to the user.

## 1 Introduction

The evaluation of the genotoxic potential of chemical substances is essential to ensure human and environmental health and is a toxicological endpoint required by EU and international regulations, such as CLP (ECHA 2017), REACH (ECHA 2017), or ICH (ICH Expert Working Group 2011).

The genotoxic hazard, which includes evaluation of mutagenicity and cytogenotoxicity (OECD, 2024), is usually assessed by different Organization of Economic Cooperation and Development (OECD) validated *in vitro* and *in vivo* tests (e.g., Ames test (OECD 2020), chromosomal aberration test (OECD 2016a, OECD 2016b)). However, the concordance of these individual tests to detect genotoxicity is relatively low, reported to be between 50-77% for in vivo tests and 59-70% for in vitro tests (E. S. Committee 2011). For that reason, it is essential to cover several different endpoints to evaluate genotoxicity. Moreover, *in vitro* approaches are often not accepted as stand-alone replacement methods for *in vivo* tests, so they are often combined with other information sources in a relevant, reliable and unbiased way (Leist et al., 2014).

When considering how best to combine different lines of evidence coming from different information sources for genotoxicity, it was clear that an Integrated Approach to Testing and Assessment (IATA) would be an excellent way to achieve this (OECD 2020). IATAs typically begin with collecting existing data and if a decision cannot be reached then new data may be generated in order to make a final decision. An IATA can integrate many different pieces of information, including physicochemical properties, *in silico* models, grouping and read-across approaches, *in vitro* methods, *in vivo* tests and human data. An Integrated Testing Strategy (ITS) is a specific kind of IATA where a structured strategy, that involves selecting and prioritizing tests based on the characteristics of the substance being assessed, is defined. In total there are five studies used in REACH to assess genotoxicity, and the REACH Annex level dictates which are used to fulfil the Standard Information Requirements (SIR) (ECHA 2017): gene mutation test in bacteria; cytogenetic assay in mammalian cells; gene mutation assay in mammalian cells; cytogenetic assay in experimental animals; and gene mutation assay in experimental animals. According to REACH, SIR for each endpoint can be obtained experimentally or by means of *in silico* methods, under Annex XI (European Chemical Agency 2024).

Among the *in silico* methods, the QSAR methodology stands out. QSAR models explore the possible relationships between structural information and the biological or physicochemical properties of the chemicals and calculate an algorithm to represent the trend(s) observed. One advantage is their broad applicability and high-throughput nature, and a large panel of human and environmental toxicology of compounds can be evaluated by means of QSARs (Cronin, 2017; Fan et al., 2018; Fjodorova et al., 2023; Sun et al., 2016; Sun et al., 2018; Toropova et al., 2023, Tratnyek et al., 2017). Nowadays, QSAR is considered an efficient alternative approach to obtain reliable activity and/or property data of chemicals, due to the reduction, or completely replacement, of *in vivo* animal experimentation, and of some *in vitro* testing. In order to be suitable for regulatory purposes, a QSAR model should follow the five principles stated by the OECD (OECD, 2014):

a. A defined endpoint
b. An unambiguous algorithm
c. A defined domain of applicability
d. Appropriate measures of goodness-of–fit, robustness and predictivity
e. A mechanistic interpretation, if possible

In the present work, we have developed a battery of QSAR models, implemented as an ITS, that covers each Annex level required by REACH to assess genotoxicity. These models have been implemented into the ProtoPRED^Ⓡ^ prediction suite ProtoQSAR S.L. (2024) (https://protopred.protoqsar.com/) as a computational workflow called GenoITS. This computational ITS allows the user to apply these QSAR models, in combination with existing experimental data, to make a final decision about the genotoxic potential of chemical substances.

## 2 Materials and methods

### 2.1 Collection and curation of experimental data

A systematic search through different scientific databases (such as QSARToolBox (ECHA 2023) or DanishQSAR (22)) was performed. In order to ensure models' reliability, the data was filtered according to the OECD guideline followed, to guarantee that the data is selected with the same experimental protocol, as shown in Table 1. In the case of mutagenicity AMES dataset, it was retrieved from Hansen et al. publication (OECD 2020).

**Table 1:** OECD test guidelines followed for each model.

Assay	OECD test guideline
Ames test	OECD: Test No 471: Bacterial reverse mutation test (OECD 2020)
*In vitro* chromosomal aberration	OECD: Test No 473: *In Vitro* Mammalian Chromosomal Aberration Test (OECD 2016a)
Hypoxanthine-guanine phosphoribosyl transferase (*Hprt*) assay	OECD: Test No 476: *In Vitro* Mammalian Cell Gene Mutation Tests using the *Hprt* and *xprt* genes (OECD 2016c)
*In vivo* micronucleus test	OECD: Test No 474: Mammalian erythrocyte micronucleus test (OECD 2016d)
Comet assay	OECD: Test No 489: *In Vivo* Mammalian Alkaline Comet Assay (OECD 2016e)

Raw collected datasets consisted of chemical structures in SMILES (Simplified Molecular-Input Line-Entry System) notation and were treated through a curation process in order to standardise the SMILES notation used, eliminate incorrect molecules, remove counterions, eliminate inorganic and organometallic compounds and manage duplicated entries -keeping only those with consistent values and removing all the inconclusive experimental results-, by using in-house scripts.

### 2.2 Calculation and selection of molecular descriptors and model development

A total of 4676 molecular descriptors for each chemical were calculated by an in-house software that calculates a wide range of descriptors of different type, including 0D, 1D and 3D descriptors from different families such as functional groups, constitutional or topological among others. Our descriptors are implemented, coming from rdkit v2021.03 (Landrum et al., 2021) and from Mordred v1.2 libraries from Python (Moriwaki et al., 2018), as well as described by R. Todeschini and V. Consonni (Todeschini and Consonni, 2009) . Once the descriptors were calculated, the descriptor values were standardized to ensure that they are similarly scaled and mean-centred in order to improve the efficiency and interpretability of some algorithms. Then, the significance of the descriptors was evaluated in two phases. First an unsupervised feature elimination step was performed to eliminate constant, infinite, highly correlated descriptors (maintaining only one descriptor if presented a correlation >90 %) and those descriptors with empty values in >15 % of the molecules. Between both phases, an imputation step was undertaken to address any potential missing values and each of the remaining datasets was split into an 80–65 %:20–35 % training set (TS) and validation set (VS) respectively by using a *k*-means method. Finally, the descriptors were subjected to a supervised feature selection step to identify which are the more informative to this property using the following algorithms: Recursive Feature Elimination (RFE); Feature importance (FI); and Permutation Importance (PI). Several groups of descriptors were selected and the final set was manually selected in basis to their performance in preliminary models (Altmann et al., 2010; Chen and Jeong, 2007; Theng and Bhoyar, 2024).

Twelve machine learning algorithms were tested in order to find the most appropriate model, including models from the lineal-like, trees and gradient boosting families, *k*-nearest neighbour and AdaBoost algorithm. All the models were performed in an in-house python-based script using the scikit-learn v1.0.2 (Pedregosa and Varoquaux, 2011), the LightGBM v3.2.1 (OECD 2023) and the XGBoost v1.5.1 (34) python packages. For each of the algorithms preliminary models were created and the final models were selected in terms of their performance both in an internal cross-validation and in the validation set.

### 2.3 Model performance evaluation

Because all the models used in this workflow were binary classification models, confusion matrices were generated in each case as a measure of their predictive power. In these matrices each row represents an actual class and each column a predicted class, thus each intersection gives information about: the positive compounds predicted as positive (True Positive, TP); the positive compounds predicted as negative (False Negative, FN); the negative compounds predicted as negative (True Negative, TN); and the negative compounds predicted as positive (False Positive, FP).

Then, a series of standard statistics are calculated using these values, the following being the most relevant:

**Accuracy.** The accuracy measures the ratio of correct predictions among the total number of predictions.

(1) $$Accuracy=\frac{TP+TN}{TP+FP+TN+FN}$$

**Sensitivity.** The sensitivity measures the proficiency of the model to correctly predict the positive experimental values.

(2) $$Sensitivity=\;\frac{TP}{TP+FN}$$

**Specificity.** The sensitivity measures the proficiency of the model to correctly predict the negative experimental values.

(3) $$Specificity=\;\frac{TN}{TN+FP}$$

Other statistics are also made available in the corresponding QMRF for every generated model in order to supply the whole picture.

### 2.4 Applicability domain

The definition -and its complexity- of the Applicability Domain (AD) is addressed by the regulators in the OECD recommendation about the development and validation of QSAR models (European Chemicals Agency 2008). Within the guideline, one of the discussed points is the different methods to evaluate the AD (Jaworska et al., 2005). There two general approaches are described: the descriptor-based methods, such as leverage, ranges and distances; and the structural similarity-based methods, which includes the Jaccard-Tanimoto coefficient, among others. In our models, four different approaches are used to calculate the AD of the models, one based on the structural fingerprints (Jaccard, 1908; Tanimoto, 1958) and the three others based on the set of molecular descriptors used to generate the model (Johnson and Wichern, 2007). In order to be considered inside the AD the compound should fulfil the requirements for at least one of the methods. Because the different approaches evaluate different aspects of the AD, they are treated independently and, information about the method(s) that considers the prediction inside the AD is given.

Tanimoto: The Jaccard-Tanimoto coefficient compares the structural similarity of two chemical structures by computing a set of MACCS fingerprints for each chemical compound and comparing them. A value from 0 to 1 is obtained, where closer to 1 corresponds to similar structures and closer to zero different ones. A threshold value of 0.528 is stipulated (Landrum, 2024). If the value between the studied molecule and any of the training dataset molecules is above this threshold the compound is inside the AD.

Euclidean distance: The Euclidean distance serves as a metric for the distance between two points within a specified space. While commonly applied in geometry with spatial coordinates, it can be extended to consider different standardized descriptor values as coordinates. We compute pairwise Euclidean distances between all the substances in the training set. In this scenario, the threshold value is determined by the maximum distance within the training set. A substance is considered to be inside the ADif its maximum distance to a training point is lower or equal.

Leverage: The leverage method uses the distance of a compound to the structural centroid of the training set as to decide if the molecule to predict lies inside the AD. The leverage value for each substance is computed using the leverage matrix, which is derived from the descriptors matrix of the entire training set (considering the entire dataset simultaneously). Given that the size of this matrix is influenced by the number of descriptors (p) and the number of substances in the training set (n), these values impact the precise leverage calculation. Therefore, the threshold value to consider a molecule within the AD based on its leverage is defined by an expression involving these numbers: 3*(p/n). The threshold is dependant of the selected set of descriptors to generate the model so, the specific value for each model is given in its QMRF.

Range: The range method relies on assessing the spread of values for each descriptor within the training set. This is achieved by contrasting the minimum and maximum values identified in the standardized descriptors. In this scenario, a substance is considered to fall within the AD if all its values lie within the established range

## 3 Results and discussion

### 3.1 Correlation analysis between experimental data from different endpoints

In order to develop QSAR models to address the different experimental studies required by REACH, we collected data from different sources, as shown in Table 2. This data is also presented in Supplementary Information as SI-1.

**Table 2:** Different studies and assays considered in the present ITS.

Study	Assay	Reference	Number of chemicals after curation
Gene mutation study in bacteria	Ames test	Hansen et al. (Hansen et al., 2009)	6492 (3492 positive & 3000 negative)
Cytogenetic study in mammalian cells	*In vitro* chromosomal aberration	OASIS database (Laboratory of Mathematical Chemistry, 2024) retrieved from QSARToolBox (https://qsartoolbox.org/)	191 (92 positive & 99 negative)
Gene mutation study in mammalian cells	*Hprt* assay	ECHA REACH (ECHA, 2024) public database retrieved from QSARToolbox	532 (56 positive & 476 negative)
Cytogenetic study in experimental animals	*In vivo* micronucleus test	ISSMIC (Istituto Superiore di Sanità, 2024) public database retrieved from QSARToolBox (https://qsartoolbox.org/)	272 (154 positive & 118 negative)
Gene mutation study in experimental animals	Comet assay	Danish (Q)SAR Database (T. U. of D 2024)	265 (127 positive & 138 negative)

**Fig. 1:**
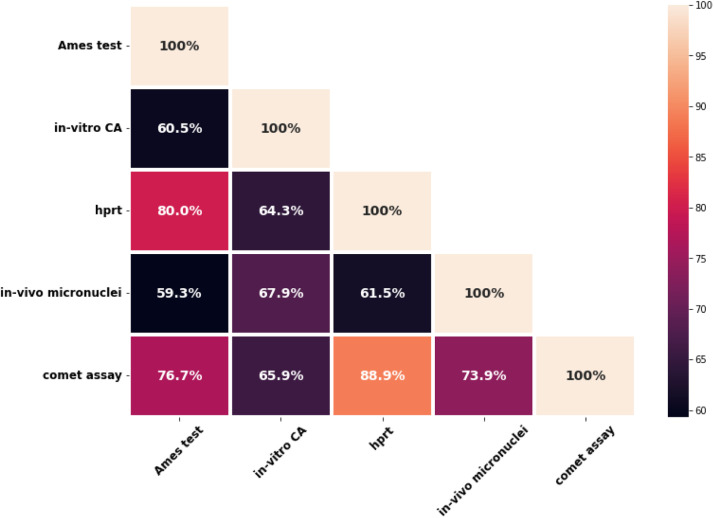
Correlation matrix (in percentages) between the experimental data available for the different assays.

Considering the abundance of available data, a first concern arises. Is it necessary to fulfil each SIR when we work with QSAR models? Or could the model developed from the *in vivo* data be sufficient to classify the compound as genotoxic/non-genotoxic? To address these questions, we have compared the collected data for the five assays: bacterial gene mutation (Ames test); *in vitro* cytogenetic assay (chromosomal aberration); *in vitro* gene mutation (*Hprt* assay); *in vivo* cytogenetic assay (*in vivo* micronucleus test); and *in vivo* gene mutation assay (comet assay). From these data we selected the common chemicals among the different assays and performed a correlation analysis (Fig. 1), showing the percentage of compounds with the same outcome value (genotoxic/non-genotoxic).

As can be seen in Fig. 1, the between-assay correlation of the common substances can be quite low, reinforcing the necessity to combine a battery of tests to make a reliable conclusion about the genotoxicity potential of chemicals.

When comparing studies measuring the same endpoint (gene mutation: Ames test, *Hprt* and comet assay or cytogenotoxicity: *in vitro* chromosomal aberration, *in vivo* micronuclei) obtained by different experimental types (in bacteria / *in vivo* / *in vitro*), in general, they present higher correlations than those assays measuring different endpoints. As example, in Fig. 1 can be observed that for comet assay -gene mutation- presents higher correlations with the other gene mutation endpoints -Ames test and *Hprt*- than with the cytogenotoxicity endpoints - *in vitro* chromosomal aberration and *in vivo* micronuclei-. This reinforces the reason that for higher tonnage substances, REACH requires a negative result in each type of study to consider a compound not-genotoxic.

### 3.2 QSAR models

We have developed five QSAR models, one to predict each study required by REACH to assess genotoxicity. Each model has been developed following the OECD principles for QSAR models (OECD, 2014).

The main features and metrics are specified in Table 3. These metrics are calculated from the confusion matrices of each model, which are present in the QMRFs (ProtoQSARa; ProtoQSARb; ProtoQSARc; ProtoQSARd; ProtoQSARe), available in the Supplementary Information as SI-2(a- e).

**Table 3:** Summary of the QSAR models.

Assay	Algorithm	Feature selection	No. descriptors	Set*	N	Metrics Accuracy/sensitivity/specifity
Mutagenicity (Ames test)	RandomForest Classifier	FI-LGBM	13	TS	4868	0.93/0.94/0.91
				VS	1624	0.76/0.78/0.74
Chromosomal aberration	LogisticRegression	RFE-LogReg	11	TS	127	0.74/0.74/0.74
				VS	64	0.70/0.70/0.71
*Hprt*	SGDClassifier	RFE-LogReg	23	TS	372	0.87/0.90/0.86
				VS	160	0.79/0.71/0.80
*In vivo* micronucleus	SVC	RFE-SVM	13	TS	203	0.75/0.76/0.73
				VS	69	0.67/0.70/0.64
Comet assay	LogisticRegression	RFE-LogReg	16	TS	185	0.92/0.94/0.90
				VS	80	0.84/0.86/0.82

⁎ TS: Train set; VS: Validation set.

Each model displays exceptionally high performance against the training set with only a small reduction in performance when assessed against the validation set. The majority of the models have an accuracy, sensitivity and specificity considerably above 0.7, with the *in vivo* micronucleus model performing only slightly lower.

Besides, in order to ensure the robustness of our models a 5-fold cross-validation has been performed using only the training set. The results are shown in Table 4. A complete compilation of the metrics of all the models can be found in Supplementary Information as SI-3.

**Table 4:** 5-fold cross-validation results of the QSAR models.

Assay	Train CV			Test CV		
	Accuracy	Sensitivity	Specificity	Accuracy	Sensitivity	Specificity
**Mutagenicity (Ames test)**	0.92 ± 0.00	0.94 ± 0.00	0.90 ± 0.00	0.78 ± 0.01	0.79 ± 0.02	0.76 ± 0.02
**Chromosomal aberration**	0.73 ± 0.03	0.73 ± 0.02	0.73 ± 0.04	0.69 ± 0.11	0.70 ± 0.17	0.68 ± 0.13
***Hprt***	0.87 ± 0.02	0.89 ± 0.05	0.87 ± 0.02	0.83 ± 0.04	0.67 ± 0.23	0.84 ± 0.04
***In vivo* micronucleus**	0.77 ± 0.02	0.80 ± 0.02	0.74 ± 0.05	0.68 ± 0.03	0.74 ± 0.04	0.58 ± 0.07
**Comet assay**	0.93 ± 0.02	0.95 ± 0.02	0.91 ± 0.02	0.88 ± 0.02	0.91 ± 0.05	0.86 ± 0.02

Furthermore, a comparison against other available free-tools have been performed -and it is supplied in the Supplementary Information (SI-4 file, Tables S1-S5). In this comparison different models coming from VEGA and DanishQSAR platforms have been analysed. From these results can be concluded that the QSAR models used by GenoITS are comparable with the others, and the information supplied in the QMRFs is, at least, the same showed in the abovementioned tools.

### 3.3 Web platform

**Fig. 2:**
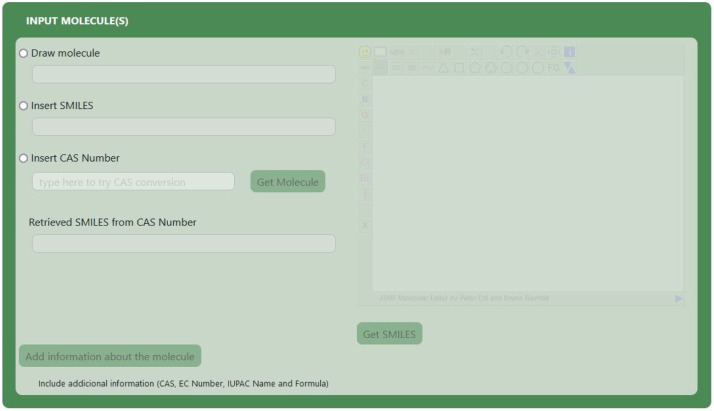
Input molecule section of GenoITS.

The aforementioned QSAR models have been individually implemented in the ProtoTOX module of the ProtoPRED^Ⓡ^ web-based tool (ProtoQSAR) (https://protopred.protoqsar.com/ProtoTOX_info).In order to facilitate access to the QSAR models, integrated as GenoITS, a specific module has been allocated in our user-friendly web platform ProtoQSAR S.L. (2024). GenoITS allows users to input their chemical in several ways; by drawing it, inputting the SMILES or inputting the CAS number, as shown in Fig. 2.

**Fig. 3:**
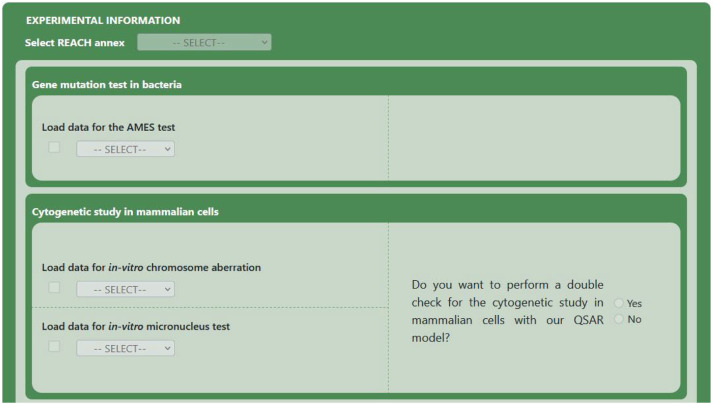
Experimental information section of GenoITS.

The platform allows users to use their own experimental data for the compound being analysed, as shown in Fig. 3. Conversely, if experimental data is not available or if the user wishes to cross-validate the provided experimental result, the platform will employ values predicted by in-house QSAR models. Therefore, for the automated predictive workflow to proceed, the user is required to provide just two essential inputs: the compound under analysis and the REACH Annex of interest.

Upon completion of the workflow, the platform furnishes the user with various outputs. These outputs consist of: multiple on-screen tables, with the first table presenting the overall result, followed by separate tables for each study utilized in the workflow, each displaying its specific outcome; a single downloadable Excel file containing the same information; a report with the general conclusion for the ITS; and the QPRF file for each QSAR prediction used during the workflow.

### 3.4 Integrated testing strategy

The web platform consists in a computational workflow able to guide the user to make decisions about the genotoxic potential of chemicals, suitable for use to fulfil REACH regulation SIRs (ECHA 2017).

The workflow considers five different types of study and specific assays for each study, as shown in Table 1. Once all the input information is supplied by the user, the first step is to handle this data in order to know when to use an in-house QSAR model. This sequential process works as follows:

1. If the user does not provide information for a specific study, the application will proceed by utilizing data from the in-house QSAR model.
2. When the compound is included in the initial dataset, the experimental value will appear instead of the predicted value.
3. If the user supplies experimental values for multiple assays within the same study, a comparison will be made between the different experimental classifications of the compound. If the classifications for a compound are not consistent, the study result -applying the precautionary principle - will be considered genotoxic (European Commission 2000).
4. If the user's data corresponds to the same assay as the in-house model, the workflow will continue with the user-supplied data rather than the model-predicted data.
5. When the user's data pertains to a different assay than the in-house models, the option to perform a double-check with the in-house model for that study is provided. If the double-check is conducted, the application will compare the user's data and GenoITS' data for the different assays for the same study. The conclusion of the study will be non-toxic only if both results are negative.
6. In situations where no experimental data is provided, and the prediction falls outside the AD, the workflow will determine if the result of that model is decisive, or if the outcome is going to be the same regardless of its result. However, if the outcome will change depending on that model, the user will receive an alert indicating that one or more models fall outside the AD, and in consequence an experimental assay for the corresponding study will be required.

Once the outcome for each study is obtained, the corresponding decisional workflow -depending the selected Annex- is applied. Noteworthy, despite there being four REACH Annexes (Annex VII-Annex X) -depending the imported and/or manufactured quantities of the chemical to be registered- the ITS only includes three workflows to apply, as Annex VIII and Annex IX have exactly the same requisites related to genotoxicity.

#### 3.4.1 Annex VII

According to REACH regulation, when registering a chemical with an intended importing or manufacturing quantity ranging from 1 to 10 t per year (Annex VII), the initial step involves evaluating the results of the gene mutation in bacteria study, also known as the Ames test.

**Fig. 4:**
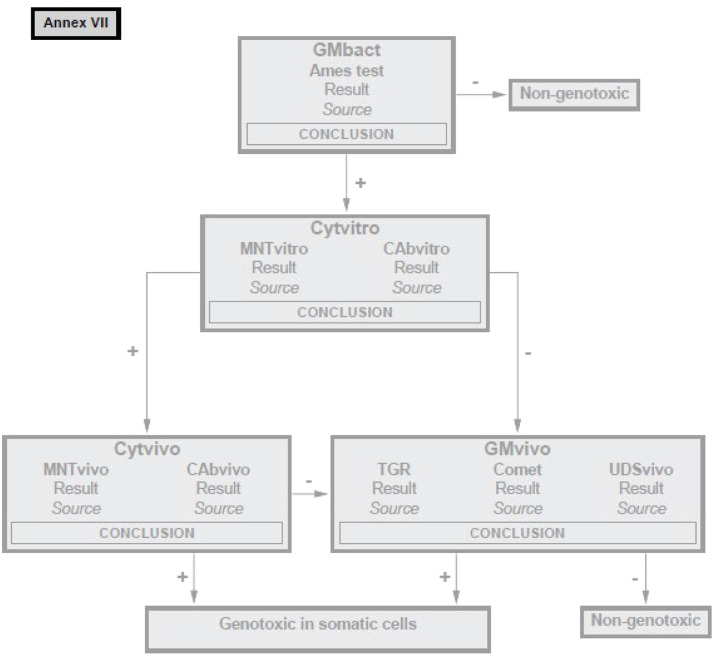
GenoITS workflow for REACH Annex VII. Abbreviations: **GMbact:** gene mutation test in bacteria; Cytvitro: cytogenetic assay in mammalian cells; CAbvitro: *in vitro* chromosome aberration test; **MNTvitro:***in vitro* micronucleus test; **GMvitro:** gene mutation assay in mammalian cells; **Cytvivo:** cytogenetic assay in experimental animals; CAbvivo: *in vivo* chromosome aberration test (bone marrow); MNTvivo: *in vivo* micronucleus test (erythrocytes); **GMvivo:** gene mutation assay in experimental animals; **UDSvivo:***in vivo* unscheduled DNA synthesis test; **TGR:***in vivo* gene mutation test with transgenic rodent; **comet:** comet assay.

As it can be seen in Fig. 4, if the Ames test yields a negative result, the compound is considered non-genotoxic. However, if the Ames test produces a positive result, an *in vitro* cytogenotoxicity study should be conducted. Ultimately, in order to categorize the compound as non-genotoxic, negative results are required in both the mutagenicity and cytogenotoxicity study categories. If the *in vitro* (or bacterial) studies demonstrate positive results, an *in vivo* version of the study should be performed.

#### 3.4.2 Annexes VIII/IX

According to REACH, when registering a chemical with an intended importing or manufacturing quantity falling between 10 and 100 tonnes per year (Annex VIII) or between 100 and 1000 tonnes per year (Annex IX), the initial step involves conducting the gene mutation in bacteria study (Ames test) and an *in vitro* cytogenotoxicity study.

**Fig. 5:**
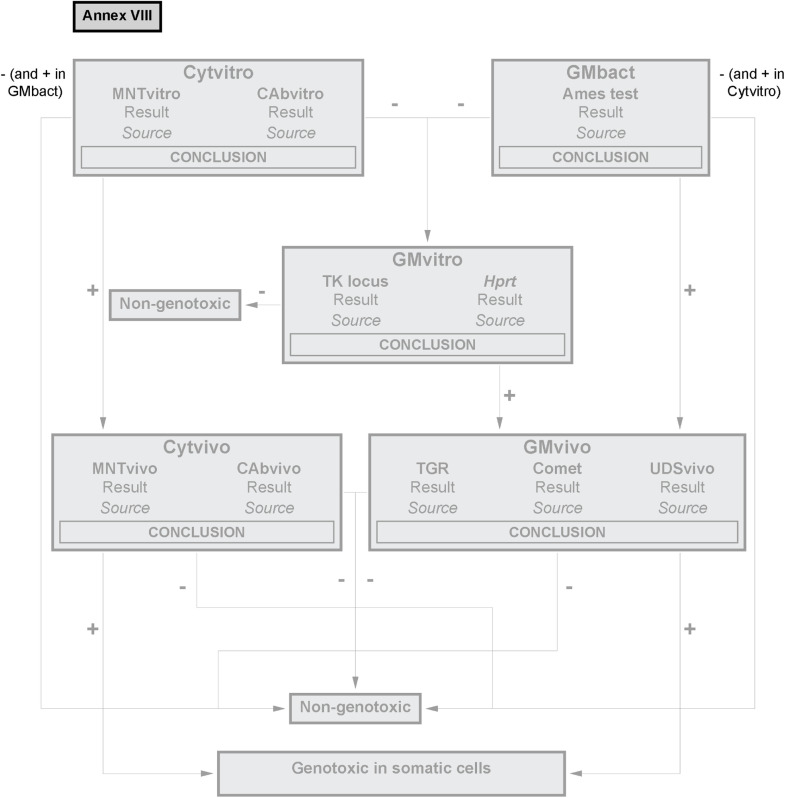
GenoITS workflow for REACH Annexes VIII and IX. Abbreviations: **GMbact:** gene mutation test in bacteria; **Cytvitro:** cytogenetic assay in mammalian cells; **CAbvitro:***in vitro* chromosome aberration test; **MNTvitro:***in vitro* micronucleus test; **GMvitro:** gene mutation assay in mammalian cells; **Cytvivo:** cytogenetic assay in experimental animals; **CAbvivo:***in vivo* chromosome aberration test (bone marrow); **MNTvivo:***in vivo* micronucleus test (erythrocytes); GMvivo: gene mutation assay in experimental animals; **UDSvivo:***in vivo* unscheduled DNA synthesis test; **TGR:***in vivo* gene mutation test with transgenic rodent; **comet:** comet assay.

As shown in Fig. 5, if both the initial studies yield negative results, and only in this scenario, a subsequent *in vitro* gene mutation study should be conducted. A negative result from this follow-up study confirms the compound as non-genotoxic.

However, if any of the previous studies produce positive results, the respective *in vivo* versions of those studies should be pursued.

To categorize a compound as non-genotoxic, it is necessary to obtain a negative conclusion in each of the study families (mutagenicity and cytogenotoxicity).

#### 3.4.3 Annex X

Like in the previous case, when registering a compound with an intended importing or manufacturing quantity exceeding 1000 tonnes per year (Annex X), the initial step involves conducting the gene mutation in bacteria study (Ames test) and an *in vitro* cytogenotoxicity study.

If both the initial studies produce negative results, and only in this circumstance, a subsequent *in vitro* gene mutation study should be conducted. If this follow-up study also yields a negative result, the compound is classified as non-genotoxic.

**Fig. 6:**
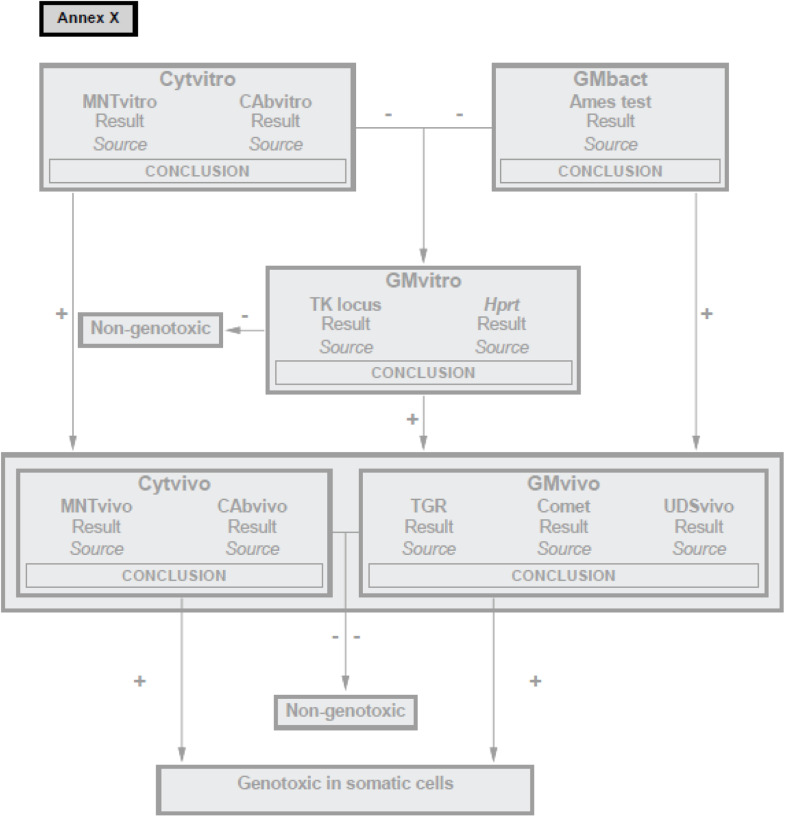
GenoITS workflow for REACH Annex X. Abbreviations: **GMbact:** gene mutation test in bacteria; Cytvitro: cytogenetic assay in mammalian cells; **CAbvitro:***in vitro* chromosome aberration test; **MNTvitro:***in vitro* micronucleus test; **GMvitro:** gene mutation assay in mammalian cells; **Cytvivo:** cytogenetic assay in experimental animals; **CAbvivo:***in vivo* chromosome aberration test (bone marrow); **MNTvivo:***in vivo* micronucleus test (erythrocytes); **GMvivo:** gene mutation assay in experimental animals; **UDSvivo:***in vivo* unscheduled DNA synthesis test; **TGR:***in vivo* gene mutation test with transgenic rodent; comet: comet assay.

However, if any of the previous studies yield positive results, further *in vivo* studies are required - as illustrated in Fig. 6. Unlike for Annex VIII and Annex IX, in this case, obtaining a negative result in one of the *in vitro* (or bacterial) studies still necessitates conducting both *in vivo* studies. Only when both *in vivo* studies yield negative results can the conclusion be drawn that the compound should be regarded as non-genotoxic. Conversely, if a positive result is obtained in either of the two *in vivo* studies, the compound is classified as genotoxic.

In all situations, a positive conclusion solely pertains to genotoxicity in somatic cells, necessitating additional tests to establish whether the compound is genotoxic in germ cells as well. Furthermore, according to REACH, a chemical classified as genotoxic is also considered to be potentially carcinogenic. Therefore, further studies in this regard are recommended for such compounds.

#### 3.4.4 Practical case

**Fig. 7:**
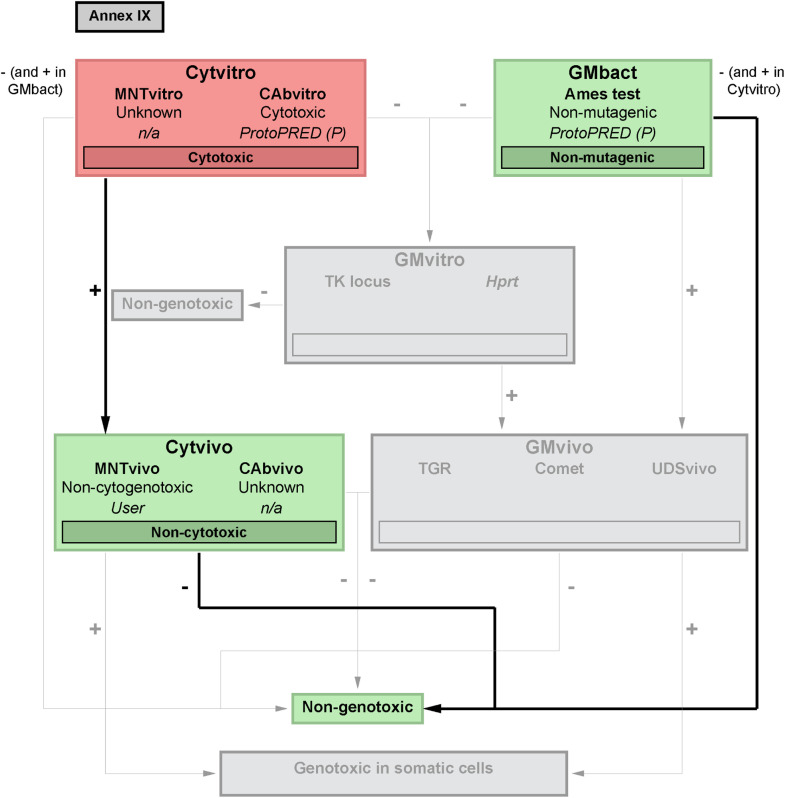
Example of the GenoITS prediction of a chemical to be registered in Annex IX. Abbreviations: **GMbact:** gene mutation test in bacteria; **Cytvitro:** cytogenetic assay in mammalian cells; **CAbvitro:***in vitro* chromosome aberration test; MNTvitro: *in vitro* micronucleus test; **GMvitro:** gene mutation assay in mammalian cells; **Cytvivo:** cytogenetic assay in experimental animals; **CAbvivo:***in vivo* chromosome aberration test (bone marrow); **MNTvivo:***in vivo* micronucleus test (erythrocytes); **GMvivo:** gene mutation assay in experimental animals; **UDSvivo:***in vivo* unscheduled DNA synthesis test; **TGR:***in vivo* gene mutation test with transgenic rodent; comet: comet assay. **ProtoPRED (P):** Value obtained by a ProtoPRED^Ⓡ^ prediction; **ProtoPRED (E):** Value coming from the experimental input datasets used in ProtoPRED^Ⓡ^.

When the web-platform is used to obtain a prediction, the schema showed above are used together with a colour protocol in order to highlight the studies analysed and their results, as observed in Fig. 7.

In Fig. 7 we can observe a colour protocol as follows:

• The studies that are not necessary according to the REACH protocol are shown in light grey, to emphasize that they are not needed.
• The boxes of the different studies are represented in green when the compound is non-toxic and in red when toxic.
• Only the final conclusion box is coloured - green if the compound is non-genotoxic, red if it's genotoxic-, the other possible final conclusions are in light grey, to represent that a conclusion cannot be reached.
• The path followed by the workflow is highlighted using dark arrows and ± symbols, all the others arrows and symbols are in light grey.
• In case the compound lies outside the applicability domain of the model applied, and the user doesn't supply own data for the study, its box will be highlighted in yellow.
• If a box of a study is highlighted in yellow, all items depending of its result will be displayed in light grey to emphasize that not decision can be taken.

This colour-coded protocol has been chosen to simplify the user's comprehension of the REACH regulation upon which the computational workflow is built.

## 4 Conclusions

Aligned with the EU recommendations concerning the refinement, reduction and replacement of *in vivo* assays used to assess the safety of chemicals for regulatory purposes, the GenoITS web platform illustrates the value and usefulness of QSARs to characterize the toxic properties of chemicals.

The SIRs required by the REACH regulation aim to optimize the available genotoxicity information of a compound. By integrating our QSAR models, the reduction of experimental tests is facilitated, resulting in time, cost, and animal savings. With or without existing experimental data, the full GenoITS workflow can be applied and a conclusion about the genotoxicity of chemicals reached without the need of additional experimental assays.

Additionally, the GenoITS web platform offers a user-friendly and intuitive graphical interface that bridges the gap between the scientific experimental community and the computationally-based methods accepted by regulatory bodies. Moreover, the GenoITS reports containing information on the chemical, the QSAR predictions (QPRFs) and the overall toxicity conclusion is designed to be used both by experts and those less familiar with in silico tools.

Finally, the web platform enables users to go beyond and make predictions for additional recommended endpoints, such as carcinogenicity, based on the results from GenoITS, within the same web platform.

Future advancements can be pursued in a similar direction. The scientific community and organisations such as the OECD have published many ITS and IATA for other endpoints such as carcinogenicity, skin and eye irritation and skin sensitization (OECD 2019; OECD 2023; Worth and Blaauboer, 2019). These existing frameworks provide opportunities for computational adaptation, serving as crucial stepping stones in the development of novel alternative methodologies for safety assessment.

## Funding statement

This project has received funding from the European Union's Horizon 2020 research and innovation programme under the Marie Skłodowska-Curie grant agreement No 101030422.

## CRediT authorship contribution statement

**José Luis Vallés-Pardo:** Writing – original draft, Software, Project administration, Methodology, Investigation, Data curation, Conceptualization. **Eva Serrano-Candelas:** Writing – review & editing, Validation, Supervision, Resources, Investigation, Data curation, Conceptualization. **Addel Goya-Jorge:** Software, Resources. **Salvador Moncho:** Writing – review & editing, Validation, Software, Resources. **Mar Crespo:** Writing – review & editing. **Donna S Macmillan:** Writing – review & editing. **Rafael Gozalbes:** Writing – review & editing, Supervision, Funding acquisition, Conceptualization.

## Declaration of competing interest

The authors declare that they have no known competing financial interests or personal relationships that could have appeared to influence the work reported in this paper.

## Data Availability

All the relevant information related to the models used to support this study are included in the QMRFs included as supplementary information files and as references within the article.

## Appendix Supplementary materials

Image, application 1

Image, application 2

Image, application 3

Image, application 4
