# Supplementary material for: GenoITS: Implementation of an Integrated Testing Strategy workflow for genotoxicity using QSAR-based tools
Source: NAM J. 2024 Dec 28;1:100005. doi: 10.1016/j.namjnl.2024.100005 (PMC13312421; doi:10.1016/j.namjnl.2024.100005)
Supplement: Supplementary file 2 [file mmc2.zip › mmc2/S2a_GenoITS_mutagenicity_ames_QMRF_report.pdf]

GenoITS

QMRF DOSSIER

ProtoQSAR model for *in vitro* gene mutation study in bacteria (Ames test)

ProtoQSAR
Computational toxicology:
fast, economical and ethical

www.protoqsar.com

Centro Europeo de Empresas Innovadoras (CEEI)
Parque Tecnológico de Valencia
Avda. Benjamin Franklin 12
46980 Paterna (Valencia, Spain)

protopred@protoqsar.com

+34 962 021 811

ProtoQSAR
Computational toxicology:
fast, economical and ethical

QMRF: ProtoQSAR model for *in vitro* gene mutation study in bacteria (Ames test) (v1.0)

1. QSAR identifier

1.1. QSAR identifier (title):

ProtoQSAR model for *in vitro* gene mutation study in bacteria (Ames test) (v1.0)

1.2. Other related models:

None

1.3. Software coding the model:

ProtoPRED® (ProtoQSAR proprietary software) v1.0

https://protoqsar.com

2. General information

2.1. Date of QMRF:

7th July 2022

2.2. QMRF author(s) and contact details:

[1] Moncho, Salvador

[2] Goya, Addel

[3] Serrano-Candelas, Eva

[4] Gozalbes, Rafael

ProtoQSAR S.L.

+34 96 202 18 11

protopred@protoqsar.com

2.3. Date of QMRF update(s):

31th July 2024

2.4. QMRF update(s):

Some texts have improved to clarify the information provided and enhance the relationship with regulatory applications

2.5. Model developer(s) and contact details:

[1] Moncho, S.

[2] Gómez-Ganau, S.

[3] Roca-Martínez, J.

[4] Barigye, S. J.

[5] Serrano-Candelas, E.

[6] Gozalbes, R.

ProtoQSAR S.L.

Contact: CEEI Valencia. Parque Tecnológico de Valencia. Avda. Benjamin Franklin 12, Desp. 28. 46980 Paterna (Valencia)
+34 96 202 18 11
protopred@protoqsar.com

2.6. Date of model development and/or publication:

February 2022

2.7. Reference(s) to main scientific papers and/or software package:

Not published.

2.8. Availability of information about the model:

The model and the algorithm are proprietary, but the dataset is non-proprietary and is available upon request.

2.9. Availability of another QMRF for exactly the same model:

Alternative QMRFs for the same model could be found in different modules of ProtoPRED (with the same content but different branding)

3. Defining the endpoint - OECD Principle 1

3.1. Species:

Bacteria (Salmonella Typhimurium)

3.2. Endpoint:

Human health effects: Mutagenicity. OECD 471: Bacterial reverse mutation test.

JRC code: QMRF 4.2. OECD test: 471

3.3. Comment on endpoint:

Mutagenicity refers to the induction of permanent transmissible changes in the amount or structure of the genetic material of cells or organisms. The Bacterial reverse mutation test evaluates gene mutations. The test uses amino-acid requiring strains of bacteria to detect (reverse) gene mutations (point mutations and frameshifts) (see reference for "Guidance on information requirements and chemical safety assessment, Chapter R.7a" in section 9.2).

The mutagenicity/genotoxicity of a substance can be estimated using different protocols. Models for *in vitro* Mammalian Chromosome Aberration Test, *Hprt* assay, *in vivo* Mammalian Erythrocyte Micronucleus test and comet assay are also available in ProtoPRED.

3.4. Endpoint units:

N/A

3.5. Dependent variable:

The dependent variable for modelling purposes is a binary classification in two categories. Original data was retrieved as a binary classification: positive (mutagenic) / negative (non-mutagenic).

3.6. Experimental protocol:

Endpoint following the OECD: Test No. 471: Bacterial reverse mutation test.

The principle of this bacterial reverse mutation test is that it detects mutations which revert mutations present in the test strains and restore the functional capability of the bacteria to synthesize an essential amino acid (histidine).

3.7. Endpoint data quality and variability:

The data for developing the model was extracted from Hansen K et al research publication. After curation and preprocessing the database is formed by 6492 experimental results, with a 53.8% of positive values (3492) and a 46.2% of negative values (3000).

4. Defining the algorithm - OECD Principle 2

4.1. Type of model:

QSAR

4.2. Explicit algorithm:

Random Forest Classifier. This method is an ensemble learning method fundamentally based on decision trees, that implements a meta estimator that fits a number of decision tree classifiers on various sub-samples of the dataset and uses averaging to improve the predictive accuracy and control over-fitting.

4.3. Descriptors in the model:

- **GATS5c:** Geary autocorrelation of lag 5 (log function) weighted by Gasteiger charge.
- **GATS1d:** Geary autocorrelation of lag 1 (log function) weighted by sigma electrons.
- **BELd-1:** Lowest eigenvalue of Burden matrix weighted by sigma electrons.
- **D/Dr3:** Distance/detour ring index of order 3.
- **SIC1:** Structural information Content index (neighborhood symmetry of 1-order).
- **N-071:** Ar-NAl2.
- **MAXDN:** Maximum of the negative delta Ii.
- **MATS1s:** Moran autocorrelation of lag 1 (log function) weighted by I-state.
- **BELc-1:** Lowest eigenvalue of Burden matrix weighted by charge.
- **BELi0:** Highest eigenvalue of Burden matrix weighted by ionization potential.
- **MIC1:** Modified information Content index (neighborhood symmetry of 1-order).
- **AATS1i:** Averaged Broto-Moreau autocorrelation of lag 1 (log function) weighted by ionization potential.
- **AATSC1i:** Averaged centred Broto-Moreau autocorrelation of lag 1 (log function) weighted by ionization potential.

4.4. Descriptor selection:

The descriptor selection is performed by eliminating non-variant descriptors and filtering collinear descriptors (R^2 > 0.9). Afterwards, the most important features were retrieved by means of gradient boosting machine.

4.5. Algorithm and descriptor generation:

Descriptors are calculated by an in-house software module in which these are implemented as described in Todeschini & Consonni, 2009 and Consonni & Todeschini, 2010 (full references in 9.2).

4.6. Software name and version for descriptor generation:

ProtoPRED® (ProtoQSAR proprietary software) v1.0

4.7. Chemicals/Descriptors ratio:

Ratio: 4868/13 = 374.46

5. Defining the applicability domain - OECD Principle 3

5.1. Description of the applicability domain of the model:

The applicability domain is defined by the training set based on several criteria:

- by chemical similarity:

Evaluated by Tanimoto-Jaccard similarity index based on molecular fingerprints (>= 0.528)

- by molecular descriptors by three different criteria:

Evaluated by the Leverage of model descriptors (<= 0.01).

Evaluated by the Euclidean distance of model descriptors (see 4.3).

Evaluated by the range of values for each descriptor.

5.2. Method used to assess the applicability domain:

- Tanimoto: The Tanimoto-Jaccard coefficient allows to compare the structural similarity of two chemical structures by computing a set of MACCS fingerprints for each chemical compound. A value from 0 to 1 is obtained, where 1 corresponds to identical structures and is closer to zero if they are very different.

- Euclidean distance: The Euclidean distance is a measure of the separation between two points in Euclidean space. We compute the distance of the descriptor values of the molecule to the descriptor values of the molecules present in the training set and determine if it is inside the applicability domain or not.

- Leverage: The leverage of a compound measures the distance of this compound to the structural centroid of the training set and is a measure of its influence on the model.

- Descriptors range: The range of standardized values for each descriptor in the train is independently evaluated by determining its maximum and minimum values. External values are standardized using the same rules and compared with the maximum and minimum value.

5.3. Software name and version for applicability domain assessment:

ProtoPRED® (ProtoQSAR proprietary software) v1.0

5.4. Limits of applicability:

The model was built only for discrete organic chemicals. A prediction is considered to fall outside the AD if it does not match any of the criteria specified in QMRF section 5.2.

6. Internal validation - OECD Principle 4

6.1. Availability of the training set:

The curated training and validation sets are not included as supporting information, but they can be provided upon request for regulatory assessment.

6.2. Available information for the training set:

- ***CAS RN:*** No
- ***Chemical Name:*** No
- ***SMILES:*** Yes
- ***Formula:*** No
- ***INChI:*** No
- ***MOL file:*** No

6.3. Data for each descriptor variable for the training set:

The descriptor values for training set are not included as supporting information.

6.4. Data for the dependent variable (response) for the training set:

The dependent variable for training set is not included as supporting information, but it is available upon request.

6.5. Other information about the training set:

The training set is comprised of 4868 (75%) compounds from a curated dataset of 6492 compounds. Compounds were selected for the training set using the Kmeans algorithm and a random split of resulting clusters, ensuring a balanced distribution on positive and negative values.

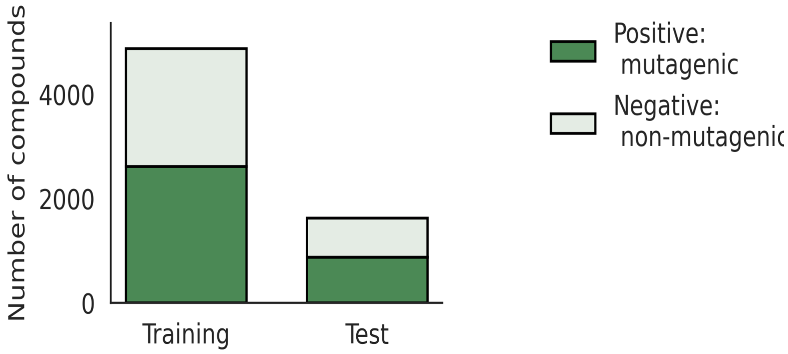

6.6. Pre-processing of data before modelling:

The experimental data of this dataset was curated following a standard procedure in order to guarantee its quality. Compounds with unclearly defined chemical structures were deleted, as well as inorganics compounds, metal complexes, salts containing organic polyatomic counterions, mixtures and substances of unknown or variable composition (UVCB). Also, duplicates and tautomers were checked.

6.7. Statistics for goodness-of-fit:

Experimental values	QSAR predictions		
	non-mutagenic	mutagenic	
**non-mutagenic**	2048	203	91.0% (TNR)
**mutagenic**	154	2463	94.0% (TPR)
	92.0 % (NPV)	92.0% (PPV)	**93.0% (ACC)**

Parameters	Training
Accuracy (ACC)	0.93
Sensitivity, recall or true positive rate (TPR)	0.94
Specificity or true negative rate (TNR)	0.91
Precision or positive predictive value (PPV)	0.92
Area under the ROC (AUC)	0.93
Negative predictive value (NPV)	0.92
F-score	0.93
Critical Success Index (CSI)	0.87
Matthews Correlation Coefficient (MCC)	0.85

6.8. Robustness – Statistics obtained by leave-one-out cross-validation:

Not reported.

6.9. Robustness – Statistics obtained by leave-many-out cross-validation:

The dataset used to train the model was divided with a (stratified) K-fold algorithm in five parts to check the robustness of the model. The average metrics of the 5 folds and their standard deviation (80% train - 20% validation) are presented here.

Parameters	Training (CV)	Validation (CV)
Accuracy (ACC)	0.92 ± 0.00	0.78 ± 0.01
Sensitivity, recall or true positive rate (TPR)	0.94 ± 0.00	0.79 ± 0.02
Specificity or true negative rate (TNR)	0.90 ± 0.00	0.76 ± 0.02
Precision or positive predictive value (PPV)	0.92 ± 0.00	0.80 ± 0.01
Area under the ROC (AUC)	0.92 ± 0.00	0.78 ± 0.01
Negative predictive value (NPV)	0.92 ± 0.00	0.80 ± 0.01
F-score	0.93 ± 0.00	0.79 ± 0.01
Critical Success Index (CSI)	0.86 ± 0.00	0.66 ± 0.01
Matthews Correlation Coefficient (MCC)	0.84 ± 0.00	0.56 ± 0.02

6.10. Robustness – Statistics obtained by Y-scrambling:

The observed values for the dataset used to train were substituted by randomized values and the model was trained again. The average metrics for 10 sets of randomized values and their standard deviation are presented here. A significant decrease in the performance of the model is an indicator of its robustness.

Parameters	Training	Y-scrambled
Accuracy (ACC)	0.93	0.93 ± 0.00
Sensitivity, recall or true positive rate (TPR)	0.94	0.96 ± 0.00
Specificity or true negative rate (TNR)	0.91	0.90 ± 0.01
Precision or positive predictive value (PPV)	0.92	0.92 ± 0.00
Area under the ROC (AUC)	0.93	0.93 ± 0.00
Negative predictive value (NPV)	0.92	0.92 ± 0.00
F-score	0.93	0.94 ± 0.00
Critical Success Index (CSI)	0.87	0.89 ± 0.01
Matthews Correlation Coefficient (MCC)	0.85	0.87 ± 0.01

6.11. Robustness – Statistics obtained by bootstrap:

Not reported.

6.12. Robustness – Statistics obtained by other methods:

Not reported.

7. External validation - OECD Principle 4

7.1. Availability of the external validation set:

The curated training and validation sets are not included as supporting information, but they can be provided upon request for regulatory assessment.

7.2. Available information for the external validation set:

- CAS RN: No

- Chemical Name: No

- SMILES: Yes

- Formula: No

- INChI: No

- MOL file: No

7.3. Data for each descriptor variable for the external validation set:

The descriptor values for validation set are not included as supporting information.

7.4. Data for the dependent variable for the external validation set:

The dependent variable for validation set is not included as supporting information, but it is available upon request.

7.5. Other information about the external validation set:

The external validation set is comprised of 1624 (25%) compounds from a curated dataset of 6492 compounds. Compounds were selected for the external validation set using the Kmeans algorithm and a random split of resulting clusters, ensuring a balanced distribution on positive and negative values.

7.6. Experimental design of test set:

Not reported.

7.7. Predictivity - Statistics obtained by external validation:

Experimental values	QSAR predictions		
	**non-mutagenic**	**mutagenic**	
**non-mutagenic**	553	196	74.0% (TNR)
**mutagenic**	190	685	78.0% (TPR)
	78.0 % (NPV)	78.0% (PPV)	**76.0% (ACC)**

Parameters	Validation
Accuracy (ACC)	0.76
Sensitivity, recall or true positive rate (TPR)	0.78
Specificity or true negative rate (TNR)	0.74
Precision or positive predictive value (PPV)	0.78
Area under the ROC (AUC)	0.76
Negative predictive value (NPV)	0.78
F-score	0.78
Critical Success Index (CSI)	0.64
Matthews Correlation Coefficient (MCC)	0.52

The full dataset of the model (including the external validation set) has been divided with a (Stratified) K-fold algorithm in five parts to check the robustness of the model. The average metrics of the 5 folds and their standard deviation (80% train - 20% validation) are presented here.

Parameters	Training (CV)	Validation (CV)
Accuracy (ACC)	0.79 ± 0.01	0.79 ± 0.01
Sensitivity, recall or true positive rate (TPR)	0.81 ± 0.02	0.81 ± 0.02
Specificity or true negative rate (TNR)	0.77 ± 0.02	0.77 ± 0.02
Precision or positive predictive value (PPV)	0.80 ± 0.02	0.80 ± 0.02
Area under the ROC (AUC)	0.79 ± 0.01	0.79 ± 0.01
Negative predictive value (NPV)	0.80 ± 0.02	0.80 ± 0.02
F-score	0.80 ± 0.01	0.80 ± 0.01
Critical Success Index (CSI)	0.67 ± 0.02	0.67 ± 0.02
Matthews Correlation Coefficient (MCC)	0.58 ± 0.03	0.58 ± 0.03

7.8. Predictivity – Assessment of the external validation set:

Among the validation set, 100.0% of the molecules are inside the applicability domain by at least one method. Particularly, 99.8% by the Tanimoto-Jaccard method, 95.9% by the leverage method and 99.9% by the Euclidean distance method.

7.9. Comments on the external validation of the model:

N/A

8. Providing a mechanistic interpretation - OECD Principle 5

8.1. Mechanistic basis of the model:

The presented model identifies chemical structural features and physicochemical properties, which during the construction of the model were found to be of relevance to *in vitro* gene mutation study in bacteria (Ames test).

8.2. A priori or a posteriori mechanistic interpretation:

A posteriori mechanistic interpretation: The identified chemical structural features and physicochemical properties may serve as starting point for a posteriori mechanistic interpretation.

8.3. Other information about the mechanistic interpretation:

N/A

9. Miscellaneous information

9.1. Comments:

All ProtoPRED models are developed to meet the OECD criteria for QSAR and are valid for regulatory purposes.

The model can be applied to estimate *in vitro* gene mutation study in bacteria (Ames test). If the bacterial mutagenicity test is negative, no further tests are required for REACH Annex VII, but

additional tests (CAbvitro (*in vitro* chromosome aberration test) or preferably a MNTvitro (*in vitro* micronucleus test), and if this is negative, a GMvitro (gene mutation assay in mammalian cell) are required for Annexes VIII, IX and X. If the bacterial test is positive, all Annexes will require complete *in vitro* testing with a CAbvitro or preferably a MNTvitro (see reference for "Guidance on information requirements and chemical safety assessment, Chapter R.7a" in section 9.2).

9.2. Bibliography:

[1] Guidance on information requirements and chemical safety assessment, Chapter R.7a: https://echa.europa.eu/documents/10162/17224/information_requirements_r7a_en.pdf

[2] OECD: Test No. 471: Bacterial Reverse Mutation Test. https://www.oecd-ilibrary.org/environment/test-no-471-bacterial-reverse-mutation-test_9789264071247-en

[3] Hansen K et al. Benchmark data set for in silico prediction of Ames mutagenicity. J Chem Inf Model. 2009 Sep;49(9):2077-81.

[4] Todeschini, R. & Consonni, V. (2009). *Molecular Descriptors for Chemoinformatics*, Wiley-VCH

[5] Consonni, V., & Todeschini, R. (2010). Molecular descriptors. In Puzyn, T., Leszczynski, J. & Cronin, M. T. (Eds.) *Recent advances in QSAR studies*(pp. 29-102). Springer

9.3. Supporting information:

Files with the training and validation datasets and other additional data can be provided upon request to regulatory agencies and institutions for assessment (a non-disclosure agreement might be needed).
