# Supplementary material for: GenoITS: Implementation of an Integrated Testing Strategy workflow for genotoxicity using QSAR-based tools
Source: NAM J. 2024 Dec 28;1:100005. doi: 10.1016/j.namjnl.2024.100005 (PMC13312421; doi:10.1016/j.namjnl.2024.100005)
Supplement: Supplementary file 2 [file mmc2.zip › mmc2/S2b_GenoITS_chromosomal_aberration_QMRF_report.pdf]

GenoITS

QMRF DOSSIER

ProtoQSAR model for *in vitro* cytogenicity study in mammalian cells (chromosomal aberration)

ProtoQSAR
Computational toxicology: fast, economical and ethical

www.protoqsar.com

Centro Europeo de Empresas Innovadoras (CEEI)
Parque Tecnológico de Valencia
Avda. Benjamin Franklin 12
46980 Paterna (Valencia, Spain)

protopred@protoqsar.com

+34 962 021 811

ProtoQSAR
Computational toxicology: fast, economical and ethical

QMRF: ProtoQSAR model for *in vitro* cytogenicity study in mammalian cells (chromosomal aberration) (v1.0)

1. QSAR identifier

1.1. QSAR identifier (title):

ProtoQSAR model for *in vitro* cytogenicity study in mammalian cells (chromosomal aberration) (v1.0)

1.2. Other related models:

None

1.3. Software coding the model:

ProtoPRED® (ProtoQSAR proprietary software) v1.0

https://protoqsar.com

2. General information

2.1. Date of QMRF:

7th July 2022

2.2. QMRF author(s) and contact details:

[1] Moncho, Salvador

[2] Goya, Addel

[3] Serrano-Candelas, Eva

[4] Gozalbes, Rafael

ProtoQSAR S.L.

+34 96 202 18 11

protopred@protoqsar.com

2.3. Date of QMRF update(s):

31th July 2024

2.4. QMRF update(s):

Some texts have improved to clarify the information provided and enhance the relationship with regulatory applications

2.5. Model developer(s) and contact details:

[1] Vallés-Pardo, J.L.

[2] Gómez-Ganau, S.

[3] Roca-Martínez, J.

[4] Barigye, S. J.

[5] Serrano-Candelas, E.

[6] Gozalbes, R.
ProtoQSAR S.L.
Contact: CEEI Valencia. Parque Tecnológico de Valencia. Avda. Benjamin Franklin 12, Desp. 28. 46980 Paterna (Valencia)
+34 96 202 18 11
protopred@protoqsar.com

2.6. Date of model development and/or publication:

January 2022

2.7. Reference(s) to main scientific papers and/or software package:

Not published.

2.8. Availability of information about the model:

The model and the algorithm are proprietary, but the dataset is non-proprietary and is available upon request.

2.9. Availability of another QMRF for exactly the same model:

Alternative QMRFs for the same model could be found in different modules of ProtoPRED (with the same content but different branding)

3. Defining the endpoint - OECD Principle 1

3.1. Species:

Chinese hamster [Chinese Hamster Lung (CHL) cell line]

3.2. Endpoint:

Human health effects: Mutagenicity/Genotoxicity. *In vitro* Mammalian Chromosome Aberration Test.

JRC code: QMRF 4.10. OECD test: 473

3.3. Comment on endpoint:

Mutagenicity refers to the induction of permanent transmissible changes in the amount or structure of the genetic material of cells or organisms. Genotoxicity is a broader term and refers to processes which alter the structure, information content or segregation of DNA and are not necessarily associated with mutagenicity. The *in vitro* mammalian chromosome aberration test is a measure of *in vitro* chromosomal mutagenicity. The test evaluates structural and numerical chromosome aberrations. The test identifies substances that induce structural chromosome aberrations in cultured mammalian established cell lines, cell strains or primary cell cultures (see reference for "Guidance on information requirements and chemical safety assessment, Chapter R.7a" in section 9.2).

The mutagenicity/genotoxicity of a substance can be estimated using different protocols. Models for Bacterial Reverse Mutation Test (Ames test), *Hprt* assay, *in vivo* Mammalian Erythrocyte Micronucleus test and comet assay are also available in ProtoPRED.

3.4. Endpoint units:

N/A

3.5. Dependent variable:

The dependent variable for modelling purposes is a binary classification in two categories. Original data was retrieved as a binary classification: positive (cytotoxic) / negative (non-cytotoxic).

3.6. Experimental protocol:

Endpoint following the OECD: Test No. 473

Cell cultures of human or other mammalian origin are exposed to the test chemical both with and without an exogenous source of metabolic activation unless cells with an adequate metabolizing capability are used. At appropriate predetermined intervals after the start of exposure of cell cultures to the test chemical, they are treated with a metaphase-arresting substance, harvested, stained and metaphase cells are analysed microscopically for the presence of chromatid-type and chromosome-type aberrations. Only test performed under S9 activation have been considered.

3.7. Endpoint data quality and variability:

The data for developing the model was extracted from OASIS Genotox Database [Chromosome aberration I subset] retrieved from QSAR Toolbox. After curation and preprocessing the database is formed by 191 experimental results, with a 48.2% of positive values (92) and a 51.8% of negative values (99).

4. Defining the algorithm - OECD Principle 2

4.1. Type of model:

QSAR

4.2. Explicit algorithm:

Logistic regression. This method, also known as logit regression, maximum-entropy classification (MaxEnt) or log-linear classifier, is a machine learning classification algorithm that is used to predict the probability of a categorical dependent variable. In this model, the probabilities describing the possible outcomes of a single trial are modeled using a logistic function.

4.3. Descriptors in the model:

- **C-015:** =CH2.
- **P-117:** X3-P=X (phosphate).
- **ATSC7i:** Centred Broto-Moreau autocorrelation of lag 7 (log function) weighted by ionization potential.
- **AATSC3dv:** Averaged centred Broto-Moreau autocorrelation of lag 3 (log function) weighted by valence electrons.
- **H8p:** H autocorrelation of lag 8 / weighted by polarizability.
- **Mor28m:** 3D MoRSE signal 28, mass-weighted .
- **Mor30v:** 3D MoRSE signal 30, van de Waals volume-weighted .
- **B02(N-O):** Presence/absence of N-O at topological distance 2.

- **B07(O-Cl):** Presence/absence of O-Cl at topological distance 7.
- **nPiperdine:** Number of piperdine rings.
- **PEOE_VSA1:** MOE Charge VSA descriptor 1.

4.4. Descriptor selection:

The descriptor selection is performed by eliminating non-variant descriptors, as well as filtering collinear descriptors (R^2 > 0.9). Afterwards, by Recursive Feature Elimination (RFE) based on a logarithmic regression, the number of descriptors was reduced based on their correlation with the values of the independent variable.

4.5. Algorithm and descriptor generation:

Descriptors are calculated by an in-house software module in which these are implemented as described in Todeschini & Consonni, 2009 and Consonni & Todeschini, 2010 (full references in 9.2).

4.6. Software name and version for descriptor generation:

ProtoPRED® (ProtoQSAR proprietary software) v1.0

4.7. Chemicals/Descriptors ratio:

Ratio: 127/11 = 11.55

5. Defining the applicability domain - OECD Principle 3

5.1. Description of the applicability domain of the model:

The applicability domain is defined by the training set based on several criteria:

- by chemical similarity:

Evaluated by Tanimoto-Jaccard similarity index based on molecular fingerprints (>= 0.528)

- by molecular descriptors by three different criteria:

Evaluated by the Leverage of model descriptors (<= 0.26).
Evaluated by the Euclidean distance of model descriptors (see 4.3).
Evaluated by the range of values for each descriptor.

5.2. Method used to assess the applicability domain:

- Tanimoto: The Tanimoto-Jaccard coefficient allows to compare the structural similarity of two chemical structures by computing a set of MACCS fingerprints for each chemical compound. A value from 0 to 1 is obtained, where 1 corresponds to identical structures and is closer to zero if they are very different.

- Euclidean distance: The Euclidean distance is a measure of the separation between two points in Euclidean space. We compute the distance of the descriptor values of the molecule to the descriptor values of the molecules present in the training set and determine if it is inside the applicability domain or not.

- Leverage: The leverage of a compound measures the distance of this compound to the structural centroid of the training set and is a measure of its influence on the model.

- ***Descriptors range:*** The range of standardized values for each descriptor in the train is independently evaluated by determining its maximum and minimum values. External values are standardized using the same rules and compared with the maximum and minimum value.

5.3. Software name and version for applicability domain assessment:

ProtoPRED® (ProtoQSAR proprietary software) v1.0

5.4. Limits of applicability:

The model was built only for discrete organic chemicals. A prediction is considered to fall outside the AD if it does not match any of the criteria specified in QMRF section 5.2.

6. Internal validation - OECD Principle 4

6.1. Availability of the training set:

The curated training and validation sets are not included as supporting information, but they can be provided upon request for regulatory assessment.

6.2. Available information for the training set:

- ***CAS RN:*** No
- ***Chemical Name:*** No
- ***SMILES:*** Yes
- ***Formula:*** No
- ***INChI:*** No
- ***MOL file:*** No

6.3. Data for each descriptor variable for the training set:

The descriptor values for training set are not included as supporting information.

6.4. Data for the dependent variable (response) for the training set:

The dependent variable for training set is not included as supporting information, but it is available upon request.

6.5. Other information about the training set:

The training set is comprised of 127 (66%) compounds from a curated dataset of 191 compounds. Compounds were selected for the training set using the Kmeans algorithm and a random split of resulting clusters, ensuring a balanced distribution on positive and negative values.

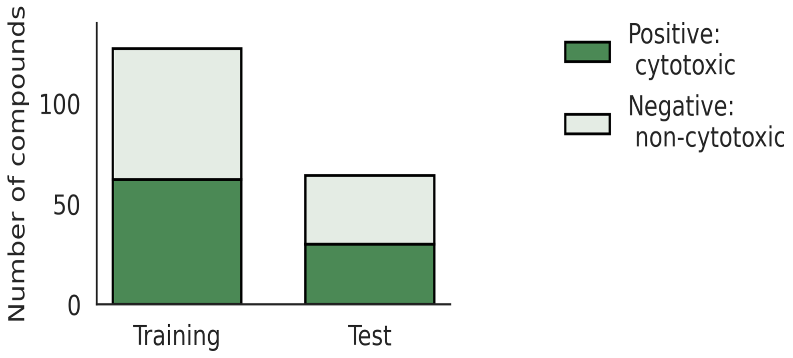

6.6. Pre-processing of data before modelling:

The experimental data of this dataset was curated following a standard procedure in order to guarantee its quality. Compounds with unclearly defined chemical structures were deleted, as well as inorganics compounds, metal complexes, salts containing organic polyatomic counterions, mixtures and substances of unknown or variable composition (UVCB). Also, duplicates and tautomers were checked.

6.7. Statistics for goodness-of-fit:

**Experimental values**	**QSAR predictions**		
	**non-cytotoxic**	**cytotoxic**	
**non-cytotoxic**	48	17	74.0% (TNR)
**cytotoxic**	16	46	74.0% (TPR)
	73.0 % (NPV)	73.0% (PPV)	**74.0% (ACC)**

**Parameters**	**Training**
Accuracy (ACC)	0.74
Sensitivity, recall or true positive rate (TPR)	0.74
Specificity or true negative rate (TNR)	0.74
Precision or positive predictive value (PPV)	0.73
Area under the ROC (AUC)	0.74
Negative predictive value (NPV)	0.73
F-score	0.74
Critical Success Index (CSI)	0.58
Matthews Correlation Coefficient (MCC)	0.48

6.8. Robustness – Statistics obtained by leave-one-out cross-validation:

Not reported.

6.9. Robustness – Statistics obtained by leave-many-out cross-validation:

The dataset used to train the model was divided with a (stratified) K-fold algorithm in five parts to check the robustness of the model. The average metrics of the 5 folds and their standard deviation (80% train - 20% validation) are presented here.

Parameters	Training (CV)	Validation (CV)
Accuracy (ACC)	0.73 ± 0.03	0.69 ± 0.11
Sensitivity, recall or true positive rate (TPR)	0.73 ± 0.02	0.70 ± 0.17
Specificity or true negative rate (TNR)	0.73 ± 0.04	0.68 ± 0.13
Precision or positive predictive value (PPV)	0.72 ± 0.04	0.68 ± 0.11
Area under the ROC (AUC)	0.73 ± 0.03	0.69 ± 0.11
Negative predictive value (NPV)	0.72 ± 0.04	0.68 ± 0.11
F-score	0.73 ± 0.03	0.68 ± 0.11
Critical Success Index (CSI)	0.57 ± 0.04	0.53 ± 0.13
Matthews Correlation Coefficient (MCC)	0.46 ± 0.06	0.39 ± 0.23

6.10. Robustness – Statistics obtained by Y-scrambling:

The observed values for the dataset used to train were substituted by randomized values and the model was trained again. The average metrics for 10 sets of randomized values and their standard deviation are presented here. A significant decrease in the performance of the model is an indicator of its robustness.

Parameters	Training	Y-scrambled
Accuracy (ACC)	0.74	0.58 ± 0.03
Sensitivity, recall or true positive rate (TPR)	0.74	0.53 ± 0.09
Specificity or true negative rate (TNR)	0.74	0.63 ± 0.11
Precision or positive predictive value (PPV)	0.73	0.58 ± 0.05
Area under the ROC (AUC)	0.74	0.58 ± 0.03
Negative predictive value (NPV)	0.73	0.58 ± 0.05
F-score	0.74	0.55 ± 0.05
Critical Success Index (CSI)	0.58	0.38 ± 0.05
Matthews Correlation Coefficient (MCC)	0.48	0.16 ± 0.07

6.11. Robustness – Statistics obtained by bootstrap:

Not reported.

6.12. Robustness – Statistics obtained by other methods:

Not reported.

7. External validation - OECD Principle 4

7.1. Availability of the external validation set:

The curated training and validation sets are not included as supporting information, but they can be provided upon request for regulatory assessment.

7.2. Available information for the external validation set:

- ***CAS RN:*** No
- ***Chemical Name:*** No
- ***SMILES:*** Yes
- ***Formula:*** No
- ***INChI:*** No
- ***MOL file:*** No

7.3. Data for each descriptor variable for the external validation set:

The descriptor values for validation set are not included as supporting information.

7.4. Data for the dependent variable for the external validation set:

The dependent variable for validation set is not included as supporting information, but it is available upon request.

7.5. Other information about the external validation set:

The external validation set is comprised of 64 (34%) compounds from a curated dataset of 191 compounds. Compounds were selected for the external validation set using the Kmeans algorithm and a random split of resulting clusters, ensuring a balanced distribution on positive and negative values.

7.6. Experimental design of test set:

Not reported.

7.7. Predictivity - Statistics obtained by external validation:

**Experimental values**	**QSAR predictions**		
	**non-cytotoxic**	**cytotoxic**	
**non-cytotoxic**	24	10	71.0% (TNR)
**cytotoxic**	9	21	70.0% (TPR)
	68.0 % (NPV)	68.0% (PPV)	**70.0% (ACC)**

Parameters	Validation
Accuracy (ACC)	0.70
Sensitivity, recall or true positive rate (TPR)	0.70
Specificity or true negative rate (TNR)	0.71
Precision or positive predictive value (PPV)	0.68
Area under the ROC (AUC)	0.70
Negative predictive value (NPV)	0.68
F-score	0.69
Critical Success Index (CSI)	0.53
Matthews Correlation Coefficient (MCC)	0.41

The full dataset of the model (including the external validation set) has been divided with a (Stratified) K-fold algorithm in five parts to check the robustness of the model. The average metrics of the 5 folds and their standard deviation (80% train - 20% validation) are presented here.

Parameters	Training (CV)	Validation (CV)
Accuracy (ACC)	0.69 ± 0.08	0.69 ± 0.08
Sensitivity, recall or true positive rate (TPR)	0.72 ± 0.06	0.72 ± 0.06
Specificity or true negative rate (TNR)	0.67 ± 0.11	0.67 ± 0.11
Precision or positive predictive value (PPV)	0.67 ± 0.10	0.67 ± 0.10
Area under the ROC (AUC)	0.69 ± 0.08	0.69 ± 0.08
Negative predictive value (NPV)	0.67 ± 0.10	0.67 ± 0.10
F-score	0.69 ± 0.08	0.69 ± 0.08
Critical Success Index (CSI)	0.54 ± 0.09	0.54 ± 0.09
Matthews Correlation Coefficient (MCC)	0.38 ± 0.17	0.38 ± 0.17

7.8. Predictivity – Assessment of the external validation set:

Among the validation set, 100.0% of the molecules are inside the applicability domain by at least one method. Particularly, 82.8% by the Tanimoto-Jaccard method, 82.8% by the leverage method and 98.4% by the Euclidean distance method.

7.9. Comments on the external validation of the model:

N/A

8. Providing a mechanistic interpretation - OECD Principle 5

8.1. Mechanistic basis of the model:

The presented model identifies chemical structural features and physicochemical properties, which during the construction of the model were found to be of relevance to *in vitro* cytogenicity study in mammalian cells (chromosomal aberration).

8.2. A priori or a posteriori mechanistic interpretation:

A posteriori mechanistic interpretation: The identified chemical structural features and physicochemical properties may serve as starting point for a posteriori mechanistic interpretation.

8.3. Other information about the mechanistic interpretation:

N/A

9. Miscellaneous information

9.1. Comments:

All ProtoPRED models are developed to meet the OECD criteria for QSAR and are valid for regulatory purposes.

The model can be applied to estimate *in vitro* cytogenicity study in mammalian cells (chromosomal aberration). A substance negative in this endpoint can be considered as non-genotoxic (*in vitro* chromosomal mutagenicity). If the test results negative and the gene mutation test in bacteria (Ames test) is negative, it is required a negative test in Mouse lymphoma assay (tk+/- locus) or hprt assay. If the test is negative and the gene mutation test in bacteria (Ames test) is positive, or the test is positive, requirements from Annex IX are required independently from the registered tonnage (see reference for "Guidance on information requirements and chemical safety assessment, Chapter R.7a" in section 9.2).

9.2. Bibliography:

[1] Guidance on information requirements and chemical safety assessment, Chapter R.7a:
https://echa.europa.eu/documents/10162/17224/information_requirements_r7a_en.pdf

[2] OECD guideline: Test No. 473: In vitro Mammalian Chromosome Aberration Test.
https://www.oecd-ilibrary.org/environment/test-no-473-in-vitro-mammalian-chromosomal-aberration-test_9789264264649-en

[3] OASIS Genotox Database
http://oasis-lmc.org/

[4] QSAR Toolbox: https://qsartoolbox.org/

[5] Todeschini, R. & Consonni, V. (2009). *Molecular Descriptors for Chemoinformatics*, Wiley-VCH

[6] Consonni, V., & Todeschini, R. (2010). Molecular descriptors. In Puzyn, T., Leszczynski, J. & Cronin, M. T. (Eds.) *Recent advances in QSAR studies*(pp. 29-102). Springer

9.3. Supporting information:

Files with the training and validation datasets and other additional data can be provided upon request to regulatory agencies and institutions for assessment (a non-disclosure agreement might be needed).
