# Supplementary material for: GenoITS: Implementation of an Integrated Testing Strategy workflow for genotoxicity using QSAR-based tools
Source: NAM J. 2024 Dec 28;1:100005. doi: 10.1016/j.namjnl.2024.100005 (PMC13312421; doi:10.1016/j.namjnl.2024.100005)
Supplement: Supplementary file 2 [file mmc2.zip › mmc2/S2c_GenoITS_hprt_assay_QMRF_report.pdf]

GenoITS

QMRF DOSSIER

ProtoQSAR model for *in vitro* gene mutation study in mammalian cells (*Hprt* assay)

ProtoQSAR
Computational toxicology: fast, economical and ethical

www.protoqsar.com

Centro Europeo de Empresas Innovadoras (CEEI)
Parque Tecnológico de Valencia
Avda. Benjamin Franklin 12
46980 Paterna (Valencia, Spain)

protopred@protoqsar.com

+34 962 021 811

ProtoQSAR
Computational toxicology: fast, economical and ethical

QMRF: ProtoQSAR model for *in vitro* gene mutation study in mammalian cells (*Hprt* assay) (v1.1)

1. QSAR identifier

1.1. QSAR identifier (title):

ProtoQSAR model for *in vitro* gene mutation study in mammalian cells (*Hprt* assay) (v1.1)

1.2. Other related models:

None

1.3. Software coding the model:

ProtoPRED® (ProtoQSAR proprietary software) v1.0

https://protoqsar.com

2. General information

2.1. Date of QMRF:

17th January 2024

2.2. QMRF author(s) and contact details:

[1] Moncho, Salvador

[2] Goya, Addel

[3] Serrano-Candelas, Eva

[4] Gozalbes, Rafael

ProtoQSAR S.L.

+34 96 202 18 11

protopred@protoqsar.com

2.3. Date of QMRF update(s):

31th July 2024

2.4. QMRF update(s):

Some texts have improved to clarify the information provided and enhance the relationship with regulatory applications

2.5. Model developer(s) and contact details:

[1] Vallés-Pardo, J.L.

[2] Serrano-Candelas, E.

[3] Gozalbes, R.

ProtoQSAR S.L.

Contact: CEEI Valencia. Parque Tecnológico de Valencia. Avda. Benjamin Franklin 12, Desp. 28. 46980 Paterna (Valencia)

+34 96 202 18 11

protopred@protoqsar.com

2.6. Date of model development and/or publication:

December 2023

2.7. Reference(s) to main scientific papers and/or software package:

Not published.

2.8. Availability of information about the model:

The model and the algorithm are proprietary, but the dataset is non-proprietary and is available upon request.

2.9. Availability of another QMRF for exactly the same model:

Alternative QMRFs for the same model could be found in different modules of ProtoPRED (with the same content but different branding)

3. Defining the endpoint - OECD Principle 1

3.1. Species:

Mouse lymphoma L5178Y cells

3.2. Endpoint:

Human health effects: Mutagenicity/Genotoxicity. *In Vitro* Mammalian Cell Gene Mutation Tests using the *Hprt* and *xprt* genes.

JRC code: QMRF 4.3. OECD test: 476

3.3. Comment on endpoint:

Mutagenicity refers to the induction of permanent transmissible changes in the amount or structure of the genetic material of cells or organisms. The purpose of the in vitro mammalian cell gene mutation test is to detect gene mutations induced by chemicals. The cell lines used in these tests measure forward mutations in reporter genes, specifically the endogenous hypoxanthine-guanine phosphoribosyl transferase gene. *Hprt*-gene mutation assay identifies substances that induce gene mutations in the *Hprt* gene of established cell lines.

The mutagenicity/genotoxicity of a substance can be estimated using different protocols. Models for Bacterial Reverse Mutation Test (Ames test), *in vitro* Mammalian Chromosome Aberration Test, *in vivo* Mammalian Erythrocyte Micronucleus test and comet assay are also available in ProtoPRED.

3.4. Endpoint units:

N/A

3.5. Dependent variable:

The dependent variable for modelling purposes is a binary classification in two categories. Original data was retrieved as a binary classification: positive (mutagenic) / negative (non-mutagenic).

3.6. Experimental protocol:

Endpoint following the OECD: Test No. 476: *In Vitro* Mammalian Cell Gene Mutation Tests using the *Hprt* and *xprt* genes

Cells in suspension or monolayer cultures are exposed to the test chemical, both with and without an exogenous source of metabolic activation, for a suitable period of time (3-6 hours), and then sub-cultured to determine cytotoxicity and to allow phenotypic expression prior to mutant selection. Cytotoxicity is determined by relative survival (RS), i.e. cloning efficiency measured immediately after treatment and adjusted for any cell loss during treatment as compared to the negative control. The treated cultures are maintained in growth medium for a sufficient period of time, characteristic of each cell type, to allow near-optimal phenotypic expression of induced mutations (typically a minimum of 7-9 days). Following phenotypic expression, mutant frequency is determined by seeding known numbers of cells in medium containing the selective agent to detect mutant colonies, and in medium without selective agent to determine the cloning efficiency (viability). After a suitable incubation time, colonies are counted. Mutant frequency is calculated based on the number of mutant colonies corrected by the cloning efficiency at the time of mutant selection.

3.7. Endpoint data quality and variability:

The data for developing the model was extracted from ECHA REACH public database retrieved from QSARToolbox. After curation and preprocessing the database is formed by 532 experimental results, with a 10.5% of positive values (56) and a 89.5% of negative values (476).

4. Defining the algorithm - OECD Principle 2

4.1. Type of model:

QSAR

4.2. Explicit algorithm:

Stochastic Gradient Descent (SGD) Classifier. This method uses an iterative optimization technique for optimizing parameters of an objective function. It is very efficient approach to fitting linear classifiers and regressors. Beginning at a particular point, the algorithm drops towards the minimum loss value by calculating the gradient and forcing the model to move in the opposite direction.

4.3. Descriptors in the model:

- **C-007:** CH2X2.
- **C-039:** Ar-C(=X)-R.
- **N-078:** Ar-N=X / X-N=X.
- **S-109:** R-SO-R.
- **ATSC4dv:** Centred Broto-Moreau autocorrelation of lag 4 (log function) weighted by valence electrons.
- **AATSC3p:** Averaged centred Broto-Moreau autocorrelation of lag 3 (log function) weighted by polarizability.

- **MATS3se:** Moran autocorrelation of lag 3 (log function) weighted by Sanderson electronegativity.
- **GATS6se:** Geary autocorrelation of lag 6 (log function) weighted by Sanderson electronegativity.
- **nR_3_True_True_None_None:** Number of 3membered rings greater fused non-aromatic and aromatic non-hetero and hetero.
- **HATS0u:** leverage-weighted autocorrelation of lag 0 / unweighted.
- **H7m:** H autocorrelation of lag 7 / weighted by mass.
- **HATS8s:** leverage-weighted autocorrelation of lag 8 / weighted by I-state.
- **Mor03m:** 3D MoRSE signal 03, mass-weighted .
- **Mor11v:** 3D MoRSE signal 11, van de Waals volume-weighted .
- **Mor22v:** 3D MoRSE signal 22, van de Waals volume-weighted .
- **Mor17s:** 3D MoRSE signal 17, I-state-weighted .
- **B05(O-O):** Presence/absence of O-O at topological distance 5.
- **nArN:** Number of N functional groups attached to aromatics.
- **nNH1:** Number of Secondary amines.
- **nPriamide:** Number of primary amides.
- **PEOE_VSA2:** MOE Charge VSA descriptor 2.
- **SLogP_VSA8:** MOE logP VSA descriptor 8.
- **RNCS:** Relative negative charge surface area.

4.4. Descriptor selection:

The descriptor selection is performed by eliminating non-variant descriptors, as well as filtering collinear descriptors (R^2 > 0.9). Afterwards, by Recursive Feature Elimination (RFE) based on a logarithmic regression, the number of descriptors was reduced based on their correlation with the values of the independent variable.

4.5. Algorithm and descriptor generation:

Descriptors are calculated by an in-house software module in which these are implemented as described in Todeschini & Consonni, 2009 and Consonni & Todeschini, 2010 (full references in 9.2).

4.6. Software name and version for descriptor generation:

ProtoPRED® (ProtoQSAR proprietary software) v1.0

4.7. Chemicals/Descriptors ratio:

Ratio: 372/23 = 16.17

5. Defining the applicability domain - OECD Principle 3

5.1. Description of the applicability domain of the model:

The applicability domain is defined by the training set based on several criteria:

- by chemical similarity:

Evaluated by Tanimoto-Jaccard similarity index based on molecular fingerprints (>= 0.528)

- by molecular descriptors by three different criteria:

Evaluated by the Leverage of model descriptors (<= 0.19).

Evaluated by the Euclidean distance of model descriptors (see 4.3).

Evaluated by the range of values for each descriptor.

5.2. Method used to assess the applicability domain:

- Tanimoto: The Tanimoto-Jaccard coefficient allows to compare the structural similarity of two chemical structures by computing a set of MACCS fingerprints for each chemical compound. A value from 0 to 1 is obtained, where 1 corresponds to identical structures and is closer to zero if they are very different.

- Euclidean distance: The Euclidean distance is a measure of the separation between two points in Euclidean space. We compute the distance of the descriptor values of the molecule to the descriptor values of the molecules present in the training set and determine if it is inside the applicability domain or not.

- Leverage: The leverage of a compound measures the distance of this compound to the structural centroid of the training set and is a measure of its influence on the model.

- Descriptors range: The range of standardized values for each descriptor in the train is independently evaluated by determining its maximum and minimum values. External values are standardized using the same rules and compared with the maximum and minimum value.

5.3. Software name and version for applicability domain assessment:

ProtoPRED® (ProtoQSAR proprietary software) v1.0

5.4. Limits of applicability:

The model was built only for discrete organic chemicals. A prediction is considered to fall outside the AD if it does not match any of the criteria specified in QMRF section 5.2.

6. Internal validation - OECD Principle 4

6.1. Availability of the training set:

The curated training and validation sets are not included as supporting information, but they can be provided upon request for regulatory assessment.

6.2. Available information for the training set:

- CAS RN: No

- Chemical Name: No

- SMILES: Yes

- Formula: No

- INChI: No

- MOL file: No

6.3. Data for each descriptor variable for the training set:

The descriptor values for training set are not included as supporting information.

6.4. Data for the dependent variable (response) for the training set:

The dependent variable for training set is not included as supporting information, but it is available upon request.

6.5. Other information about the training set:

The training set is comprised of 372 (70%) compounds from a curated dataset of 532 compounds. Compounds were selected for the training set using the Kmeans algorithm and a random split of resulting clusters, ensuring a balanced distribution on positive and negative values.

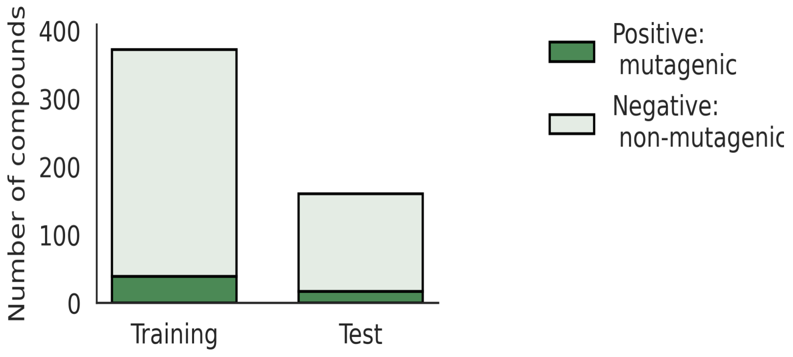

6.6. Pre-processing of data before modelling:

The experimental data of this dataset was curated following a standard procedure in order to guarantee its quality. Compounds with unclearly defined chemical structures were deleted, as well as inorganics compounds, metal complexes, salts containing organic polyatomic counterions, mixtures and substances of unknown or variable composition (UVCB). Also, duplicates and tautomers were checked.

6.7. Statistics for goodness-of-fit:

**Experimental values**	**QSAR predictions**		
	**non-mutagenic**	**mutagenic**	
**non-mutagenic**	283	50	85.0% (TNR)
**mutagenic**	4	35	90.0% (TPR)
	41.0 % (NPV)	41.0% (PPV)	**85.0% (ACC)**

Parameters	Training
Accuracy (ACC)	0.85
Sensitivity, recall or true positive rate (TPR)	0.90
Specificity or true negative rate (TNR)	0.85
Precision or positive predictive value (PPV)	0.41
Area under the ROC (AUC)	0.87
Negative predictive value (NPV)	0.41
F-score	0.56
Critical Success Index (CSI)	0.39
Matthews Correlation Coefficient (MCC)	0.55

6.8. Robustness – Statistics obtained by leave-one-out cross-validation:

Not reported.

6.9. Robustness – Statistics obtained by leave-many-out cross-validation:

The dataset used to train the model was divided with a (stratified) K-fold algorithm in five parts to check the robustness of the model. The average metrics of the 5 folds and their standard deviation (80% train - 20% validation) are presented here.

Parameters	Training (CV)	Validation (CV)
Accuracy (ACC)	0.85 ± 0.04	0.83 ± 0.06
Sensitivity, recall or true positive rate (TPR)	0.87 ± 0.02	0.72 ± 0.17
Specificity or true negative rate (TNR)	0.85 ± 0.04	0.84 ± 0.06
Precision or positive predictive value (PPV)	0.42 ± 0.08	0.37 ± 0.11
Area under the ROC (AUC)	0.86 ± 0.03	0.78 ± 0.09
Negative predictive value (NPV)	0.42 ± 0.08	0.37 ± 0.11
F-score	0.56 ± 0.07	0.47 ± 0.11
Critical Success Index (CSI)	0.39 ± 0.07	0.32 ± 0.09
Matthews Correlation Coefficient (MCC)	0.53 ± 0.07	0.43 ± 0.13

6.10. Robustness – Statistics obtained by Y-scrambling:

The observed values for the dataset used to train were substituted by randomized values and the model was trained again. The average metrics for 10 sets of randomized values and their standard deviation are presented here. A significant decrease in the performance of the model is an indicator of its robustness.

Parameters	Training	Y-scrambl ed
Accuracy (ACC)	0.85	0.60 ± 0.09
Sensitivity, recall or true positive rate (TPR)	0.90	0.65 ± 0.13
Specificity or true negative rate (TNR)	0.85	0.60 ± 0.11
Precision or positive predictive value (PPV)	0.41	0.16 ± 0.03
Area under the ROC (AUC)	0.87	0.62 ± 0.05
Negative predictive value (NPV)	0.41	0.16 ± 0.03
F-score	0.56	0.26 ± 0.04
Critical Success Index (CSI)	0.39	0.15 ± 0.02
Matthews Correlation Coefficient (MCC)	0.55	0.16 ± 0.06

6.11. Robustness – Statistics obtained by bootstrap:

Not reported.

6.12. Robustness – Statistics obtained by other methods:

Not reported.

7. External validation - OECD Principle 4

7.1. Availability of the external validation set:

The curated training and validation sets are not included as supporting information, but they can be provided upon request for regulatory assessment.

7.2. Available information for the external validation set:

- *CAS RN:* No

- *Chemical Name:* No

- *SMILES:* Yes

- *Formula:* No

- *INChI:* No

- *MOL file:* No

7.3. Data for each descriptor variable for the external validation set:

The descriptor values for validation set are not included as supporting information.

7.4. Data for the dependent variable for the external validation set:

The dependent variable for validation set is not included as supporting information, but it is available upon request.

7.5. Other information about the external validation set:

The external validation set is comprised of 160 (30%) compounds from a curated dataset of 532 compounds. Compounds were selected for the external validation set using the Kmeans algorithm and a random split of resulting clusters, ensuring a balanced distribution on positive and negative values.

7.6. Experimental design of test set:

Not reported.

7.7. Predictivity - Statistics obtained by external validation:

Experimental values	QSAR predictions		
	**non-mutagenic**	**mutagenic**	
**non-mutagenic**	108	35	76.0% (TNR)
**mutagenic**	5	12	71.0% (TPR)
	26.0 % (NPV)	26.0% (PPV)	**75.0% (ACC)**

**Parameters**	**Validation**
Accuracy (ACC)	0.75
Sensitivity, recall or true positive rate (TPR)	0.71
Specificity or true negative rate (TNR)	0.76
Precision or positive predictive value (PPV)	0.26
Area under the ROC (AUC)	0.73
Negative predictive value (NPV)	0.26
F-score	0.37
Critical Success Index (CSI)	0.23
Matthews Correlation Coefficient (MCC)	0.31

The full dataset of the model (including the external validation set) has been divided with a (Stratified) K-fold algorithm in five parts to check the robustness of the model. The average metrics of the 5 folds and their standard deviation (80% train - 20% validation) are presented here.

**Parameters**	**Training (CV)**	**Validation (CV)**
Accuracy (ACC)	0.80 ± 0.06	0.80 ± 0.06
Sensitivity, recall or true positive rate (TPR)	0.75 ± 0.16	0.75 ± 0.16
Specificity or true negative rate (TNR)	0.80 ± 0.06	0.80 ± 0.06
Precision or positive predictive value (PPV)	0.33 ± 0.11	0.33 ± 0.11
Area under the ROC (AUC)	0.78 ± 0.10	0.78 ± 0.10
Negative predictive value (NPV)	0.33 ± 0.11	0.33 ± 0.11
F-score	0.45 ± 0.13	0.45 ± 0.13
Critical Success Index (CSI)	0.30 ± 0.11	0.30 ± 0.11
Matthews Correlation Coefficient (MCC)	0.40 ± 0.16	0.40 ± 0.16

7.8. Predictivity – Assessment of the external validation set:

Among the validation set, 100.0% of the molecules are inside the applicability domain by at least one method. Particularly, 90.0% by the Tanimoto-Jaccard method, 91.2% by the leverage method and 100.0% by the Euclidean distance method.

7.9. Comments on the external validation of the model:

N/A

8. Providing a mechanistic interpretation - OECD Principle 5

8.1. Mechanistic basis of the model:

The presented model identifies chemical structural features and physicochemical properties, which during the construction of the model were found to be of relevance to *in vitro* gene mutation study in mammalian cells (*Hprt* assay).

8.2. A priori or a posteriori mechanistic interpretation:

A posteriori mechanistic interpretation: The identified chemical structural features and physicochemical properties may serve as starting point for a posteriori mechanistic interpretation.

8.3. Other information about the mechanistic interpretation:

N/A

9. Miscellaneous information

9.1. Comments:

All ProtoPRED models are developed to meet the OECD criteria for QSAR and are valid for regulatory purposes.

The model can be applied to estimate *in vitro* gene mutation study in mammalian cells (*Hprt* assay). An *in vitro* gene mutation study in mammalian cells is the second part of the standard information set required for registration at the Annex VIII tonnage level. For substances that have been tested already, this information should always be presented as part of the overall Weight of Evidence for mutagenicity with reference to induction of gene mutations in mammalian cells. For other substances, this second *in vitro* mammalian cell test will normally only be required when the results of the bacterial gene mutation test and the first study in mammalian cells (i.e. an *in vitro* chromosome aberration test or an *in vitro* micronucleus test) are negative. This is to detect in vitro mutagens that give negative results in the other two tests. (see reference for "Guidance on information requirements and chemical safety assessment, Chapter R.7a" in section 9.2)

9.2. Bibliography:

[1] Guidance on information requirements and chemical safety assessment, Chapter R.7a:
https://echa.europa.eu/documents/10162/17224/information_requirements_r7a_en.pdf

[2] OECD guideline: OECD: Test No. 476: *In Vitro* Mammalian Cell Gene Mutation Tests using the *Hprt* and xprt genes. https://www.oecd.org/chemicalsafety/test-no-476-in-vitro-mammalian-cell-gene-mutation-tests-using-the-hprt-and-xprt-genes-9789264264809-en.htm

[3] ECHA REACH database. https://echa.europa.eu/es/information-on-chemicals/registered-substances

[4] QSAR Toolbox: https://qsartoolbox.org/

[5] Todeschini, R. & Consonni, V. (2009). *Molecular Descriptors for Chemoinformatics*, Wiley-VCH

[6] Consonni, V., & Todeschini, R. (2010). Molecular descriptors. In Puzyn, T., Leszczynski, J. & Cronin, M. T. (Eds.) *Recent advances in QSAR studies*(pp. 29-102). Springer

9.3. Supporting information:

Files with the training and validation datasets and other additional data can be provided upon request to regulatory agencies and institutions for assessment (a non-disclosure agreement might be needed).
